# Neuroprotective Effects of VEGF in the Enteric Nervous System

**DOI:** 10.3390/ijms23126756

**Published:** 2022-06-17

**Authors:** Ines Hecking, Lennart Norman Stegemann, Verena Theis, Matthias Vorgerd, Veronika Matschke, Sarah Stahlke, Carsten Theiss

**Affiliations:** 1 Department of Cytology, Institute of Anatomy, Ruhr-University Bochum, Universitaetsstr. 150, Building MA, Level 5, 44780 Bochum, Germany; ines.hecking@rub.de (I.H.); lennart.stegemann@rub.de (L.N.S.); verena.theis@rub.de (V.T.); veronika.matschke@rub.de (V.M.); sarah.stahlke@rub.de (S.S.); 2 Neuromuscular Center Ruhrgebiet, Department of Neurology, University Hospital Bergmannsheil, Ruhr-University Bochum, Buerkle-de-la-Camp-Platz 1, 44789 Bochum, Germany; matthias.vorgerd@bergmannsheil.de

**Keywords:** VEGF, VEGFR-2, KDR, enteric nervous system, myenteric plexus, neurodegenerative diseases, Parkinson’s disease, gut–brain axis

## Abstract

Although the enteric nervous system (ENS) functions largely autonomously as part of the peripheral nervous system (PNS), it is connected to the central nervous system (CNS) via the gut–brain axis. In many neurodegenerative diseases, pathological changes occur in addition to gastrointestinal symptoms, such as alpha-synuclein aggregates in Parkinson’s disease, which are found early in the ENS. In both the CNS and PNS, vascular endothelial growth factor (VEGF) mediates neuroprotective and neuroregenerative effects. Since the ENS with its close connection to the microbiome and the immune system is discussed as the origin of neurodegenerative diseases, it is necessary to investigate the possibly positive effects of VEGF on enteric neurons. Using laser microdissection and subsequent quantitative RT-PCR as well as immunohistochemistry, for the first time we were able to detect and localize VEGF receptor expression in rat myenteric neurons of different ages. Furthermore, we demonstrate direct neuroprotective effects of VEGF in the ENS in cell cultures. Thus, our results suggest a promising approach regarding neuroprotection, as the use of VEGF (may) prevent neuronal damage in the ENS.

## 1. Introduction

The coordination of complex processes such as muscle activity, blood flow and absorption in the gastrointestinal tract is mediated by the largely autonomous enteric nervous system (ENS). Despite belonging to the peripheral nervous system (PNS), the ENS is referred to as the “second brain” due to its numerous similarities to the central nervous system (CNS), regarding neurotransmitters and complexity [1]. Several hundred million neurons are organized in two interconnected plexuses [2]. The myenteric plexus (Auerbach plexus) is located between the circular muscle layer and the longitudinal muscle layer, while the plexus (Meissner plexus) lies in the submucosa [3]. The neurons in both plexuses change their activity reflexively in response to ubiquitous stimuli in the gut such as stretching, mechanical deformation and altered luminal chemistry [4]. The lifelong plasticity of the ENS enables neurons and glial cells to continuously respond to dietary habits, the environment and intestinal diseases [5]. In addition to gastrointestinal diseases, the ENS is also involved in the development of diseases of other organs and systems such as the CNS.

The gut is in close bidirectional contact with the CNS both via anatomical structures such as the vagus nerve and via hormones [6,7]. The gut–brain axis seems to be the prerequisite for a functional gut microbiota and plays a crucial role in the pathogenesis of neurodegenerative diseases such as Parkinson’s and Alzheimer’s disease (AD). Various studies have shown a correlation between a dysfunctional microbiota and neurodegenerative diseases such as Parkinson’s disease (PD) [7,8]. Thus, in many neurodegenerative diseases, pathological changes in the ENS are found alongside gastrointestinal symptoms. Neurotropic factors, which are thought to trigger the alpha-synuclein aggregations typical of PD, have easy access to neurons via the gastric mucosa and can subsequently influence the brain via the gut–brain axis [9]. The ENS also appears to be involved in the development of AD. Alterations in RNA and protein expression and microglia as well as high levels of amyloid-β-peptides (Aβ) have been detected in the ENS of the small intestine in AD mouse models [10,11].

A major problem in the therapy of neurodegenerative diseases is the usually late diagnosis. Given that the ENS is easy to access for biopsies via colonoscopy, it could enable early diagnosis by demonstrating the neuronal changes typical of the respective disease [12,13]. Ultimately, therapeutic measures at the ENS may then contain or even prevent the CNS infection. For this purpose, we examine the effects of VEGF on enteric neurons in this study.

The vascular endothelial growth factor (VEGF) was first discovered as a non-toxic stimulator of vascular permeability for blood vessels [14]. Later, numerous other functions were ascertained. VEGF mediates vasculogenesis and angiogenesis, and is therefore closely connected to tumor growth [15,16]. In the CNS and PNS, the molecule is involved in neuroprotective and neuroregenerative mechanisms [17].

In mammals, the VEGF family members VEGF-A (also referred as VEGF), VEGF-B, VEGF-C, VEGF-D, and PIGF (placenta growth factor) are found [18,19,20]. Alternative splicing of the VEGF gene creates nine different isoforms of which VEGF-A-165a and VEGF-A-165b are the predominant isoforms [21,22].

While VEGF-A-165a shows neuroprotective effects and additionally stimulates angiogenesis, permeability and vasculogenesis via VEGF-receptor 2 (VEGFR-2), VEGF-A-165b binds to the receptor with the same affinity, but inhibits its signaling pathways [17,23,24]. VEGF-A-165b does not act angiogenic, but still mediates neuroprotective and neurotrophic effects in the CNS and PNS, and therefore it may be used in the therapy of neurodegenerative diseases without stimulating vasculogenesis [24,25]. Even the application of VEGF-B shows neuroprotective effects in an in vitro model of PD [26]. Such a therapeutic application of VEGF in PD has been discussed for some time. In the CNS, VEGF administration promotes neuronal survival by suppressing the neurotoxin 6-hydroxydopamine, which is involved in the pathogenesis of PD [27]. In the context of AD, Aβ acts as an antagonist of VEGFR-2, which means that pro-angiogenic therapy with VEGF is being discussed as a therapeutic option here [28].

As already described for the adult brain, it is necessary to identify and stimulate different processes involved in neurorepair like angiogenesis, neurogenesis, synaptic plasticity and endogenous neurorepair phenomena in the ENS [29]. The pro-angiogenic effects of VEGF could also have a positive impact vascular dementia as systemic small vessel diseases are thought to promote dementia and extrapyramidal symptoms and probably also affect the gut-brain axis [30].

The biological functions of VEGF are mediated via type III receptor tyrosine kinases (RTKs) VEGFR-1 (Flt-1), VEGFR-2 (KDR, Flk-1), and VEGFR-3 (Flt-4). Beside these main-receptors, VEGF also connects to a family of VEGFR-coreceptors of the neuropilin-family (NRP1 and NRP2) [19]. VEGF-A connects particularly to VEGFR-1, VEGFR-2, NRP1 and NRP2, while PIGF such as VEGF-B use VEGFR-1 and NRP1. Both VEGF-C and VEGF-D bind to VEGFR-2 and VEGFR-3, as well as to coreceptors NRP1 and NRP2 [15,31,32].

Stimulation of the VEGFR-2 signal transduction pathway leads to enhanced vascular permeability, angiogenesis, neuronal outgrowth and neuroprotection [17,19,33,34]. The functionality of VEGFR-2 is limited by the decoy receptor VEGFR-1, although the independent activities of VEGFR-1 are not fully understood [19,20]. VEGFR-3, which is mainly known for its lymphangiogenesis-promoting effects, regulates neuronal development and adult neuronal function in the CNS [35,36]. NRPs play an important role in angiogenesis and axonal guidance [37]. To investigate the effects of VEGF in the ENS, a detailed examination of the expressed VEGF receptors is required first. For studies on the possible therapeutic impact of VEGF, data on the expression of the receptors are particularly important in older age stages, since neurodegenerative diseases mostly occur in old age. Interestingly, VEGFR-2 is less strong expressed in Purkinje cells of the CNS in older age stages [38]. Different expression patterns were also found for VEGFR-3 in individual neuronal populations in the CNS [36].

The present study provides for the first time a detailed description of VEGF receptor distribution patterns in neonatal (p9), juvenile (p15), and mature (p30) enteric neurons. Laser microdissection (LMD) samples followed by quantitative reverse transcription polymerase chain reaction (qRT-PCR) and immunohistochemistry of rat small intestine were used for this purpose. In addition, the pro-survival effect of VEGF on the ENS was demonstrated in a cell culture PD model of myenteric neurons. The rotenone model established for PD was used for this purpose [26,39,40,41,42,43,44,45]. Due to its high lipophilicity, the pesticide rotenone can easily penetrate the cells, where it causes an interruption of the mitochondrial respiratory chain [39]. Long-term exposure to pesticides that impair mitochondrial functions or increase oxidative stress in the cell has been associated with an increased prevalence of PD [44,46]. In our study, concomitant administration of VEGF was performed to test its neuroprotective effect on stressed neurons.

## 2. Results

### 2.1. VEGF-Receptor Expression Pattern

**Figure 1:**
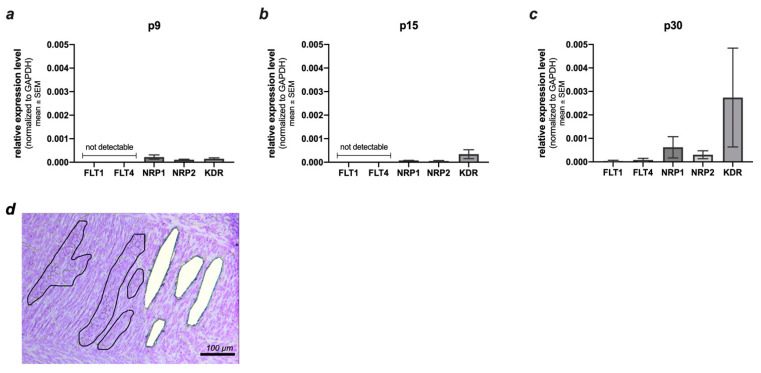
mRNA expression of VEGF receptors in the lasered myenteric plexus at p9, p15, p30. (**a**–**c**) mRNA expression levels of FLT1 (VEGFR-1); KDR (VEGFR-2); FLT4 (VEGFR-3); NRP1 and NRP2 at p9 (**a**), p15 (**b**), p30 (**c**). FLT-1 (VEGFR-1) and FLT4 (VEGFR-3) were only detected at p30 (**c**). No significant age dependent differences of the mRNA levels of KDR (VEGFR-2), NRP1 and NRP-2 were seen. (**d**) 10 μm thick cryosections of the tunica muscularis in rat small intestine (p15) stained with 0.5% cresyl violet solution. The myenteric ganglia (black line) are precisely marked and lasered out using Leica LMD6500 laser microdissection device.

To investigate the neuroprotective effects of VEGF on enteric neurons, a detailed knowledge of the receptor expression pattern was indispensable. By using laser microdissection, we were able to isolate myenteric ganglia from cresyl violet stained cryosections of rat small intestine. In subsequent qRT-PCR the mRNA expression levels of all VEGF-receptors (FLT1 (codes for VEGFR-1), FLT4 (codes for VEGFR-3), NRP1, NRP2 and KDR (codes for VEGFR-2),) were analyzed in neonatal (p9), juvenile (p15), and mature (p30) enteric neurons (Figure 1a–c). For each condition, an area of at least 21,000,000 μm^2^ was dissected, exemplified in Figure 1d. Per age stage, the small intestine from 15 rats was used. Figure 1a–c shows that FLT1 (VEGFR-1) and FLT4 (VEGFR-3) could not be detected until p30. Whereas mRNA levels of KDR (VEGFR-2) and the two co-receptors NRP1/NRP2 were expressed at all examined ages, with no significant age-dependent regulation. However, the expression of KDR increases slightly with age (p9: 1.17 × 10^−4^ (±0.38 × 10^−4^); p15: 6.04 × 10^−4^ (±2.78 × 10^−4^); p30: 24.89 × 10^−4^ (±15.66 × 10^−4^)), being the predominant receptor at p15 and p30. The expression of NRP1 (p9: 2.04 × 10^−4^ (±0.95 × 10^−4^); p15: 0.70 × 10^−4^ (±0.05 × 10^−4^); p30: 6.20 × 10^−4^ (±4.49 × 10^−4^)) and NRP2 (p9: 0.98 × 10^−4^ (±0.19 × 10^−4^); p15: 0.50 × 10^−4^ (±0.22 × 10^−4^); p30: 3.08 × 10^−4^ (±1.68 × 10^−4^)) fluctuates, as it first decreases and then marginally increases again.

### 2.2. VEGFR-2 Localization in Rat Small Intestine

**Figure 2:**
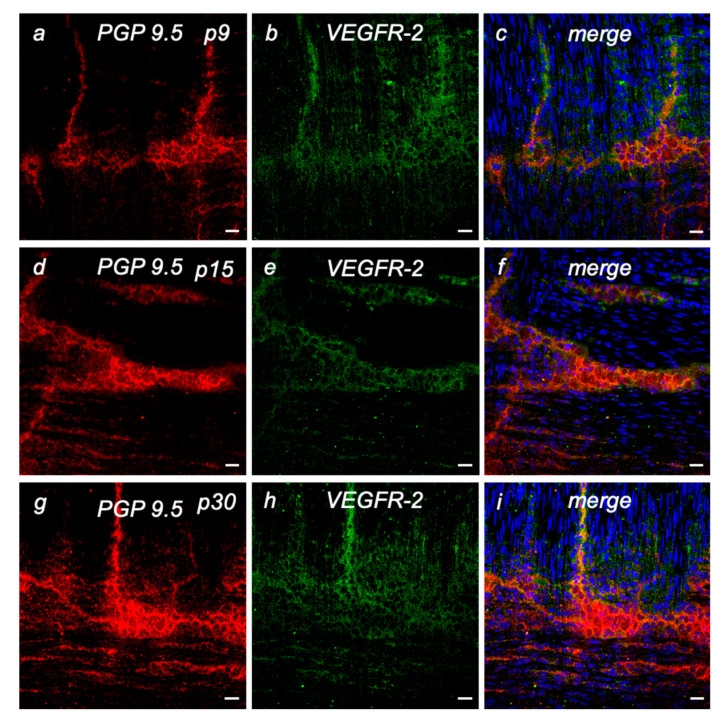
(**a**–**i**) VEGFR-2 expression in cryosections of p9, p15, and p30 rat small intestine. Immunostaining of PGP 9.5-positive neurons of the myenteric plexus (red), VEGFR-2 (green) and cell nuclei (blue). Myenteric neurons are particularly visible between the longitudinal and circular muscle layers. VEGFR-2 is predominantly found in cell bodies and cell processes of these myenteric neurons at all age examined. The same exposure settings on the confocal laser scanning microscope were used for all images. Scale bars: 20 μm.

Since the neuroprotective effect of VEGF is mediated in particular via VEGFR-2 [17,19,33,34], local expression in the intestine was also investigated in more detail in addition to the mRNA expression studies. Using immunohistochemistry, we were able to detect specific VEGFR-2 protein expression in ENS neurons (Figure 2). Neurons were stained with antibodies against PGP9.5 (Figure 2a,d,g) or TUJ-1 (Supplementary Data) as neuronal markers. Specific expression of VEGFR-2 (Figure 2b,e,h) in the myenteric neurons was evident at all ages examined (p9, p15 and p30), with VEGFR-2 expression in the somata and cell prolongations.

In summary, therefore, in agreement with the results of the qRT-PCR mRNA expression studies, VEGFR-2 can be detected in myenteric neurons at all stages examined, without any obvious age-dependent changes in expression.

### 2.3. Neuroprotective Effects of VEGF on Rotenone Treated Myenteric Neurons

Myenteric neurons from the intestine were extracted and cultured, and then treated in vitro with 800 nM rotenone for 24 h on day nine. Half of the samples were also treated simultaneously with 0.1 μg/mL VEGF.

**Figure 3:**
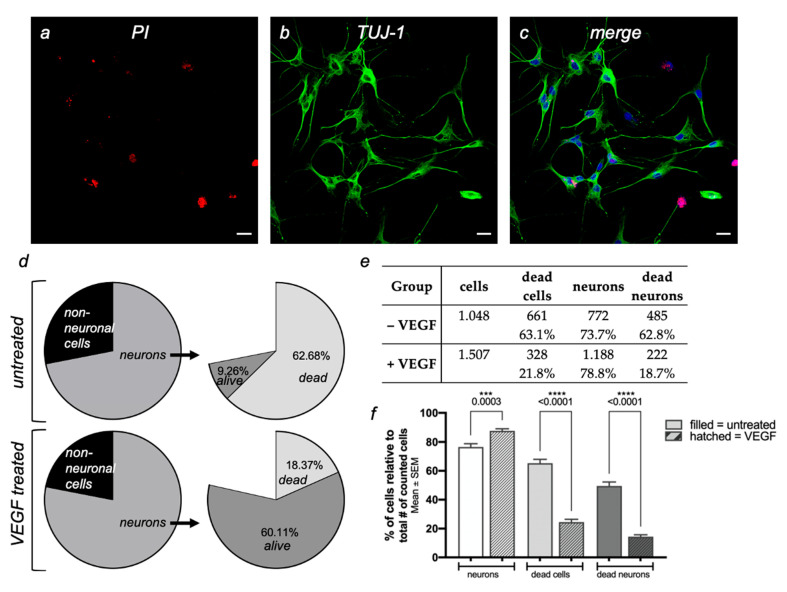
The neuroprotective effect of VEGF on cultured myenteric plexus neurons during exposition to 800 nM rotenone for 24 h. (**a**–**c**) Representative images of cultured myenteric plexus neurons. Dead cells are identified by staining with propidium iodide (red), neurons are stained with TUJ-1 (green), and cell nuclei of all cells are visualized with DAPI (blue). Scale bars: 20 μm. (**d**–**f**) Significantly more cells survived in the group with rotenone and simultaneous VEGF treatment (+VEGF) than with rotenone treatment alone (−VEGF). *** *p* < 0.001; **** *p* < 0.0001.

Immunohistochemical labelling of the samples with TUJ-1 (neuronal marker), propidium iodide (PI; necrotic cells), and DAPI (nuclear stain) was performed to determine the percentage of neurons destroyed by rotenone treatment (Figure 3a–c).

Quantitative evaluation was performed independently by two blinded individuals. This showed a significantly increased survival rate of neurons with rotenone plus VEGF treatment (Figure 3d–f). After rotenone treatment but without VEGF, 661 of 1048 cells died, which corresponds to a proportion of 63.1%. Of the neurons evaluated (772), 458 died, which again corresponds to an almost equal proportion of 62.8%. In the VEGF-treated samples, on the other hand, the proportion of cells that died as a result of rotenone treatment was significantly lower, as was the proportion of neurons. Out of 1507 cells in total, 328 died, which corresponds to a share of 21.8%. Focusing on the neurons, 222 of 1188 ENS neurons died, which again corresponds to a share of 18.7%. It is thus very clear that VEGF given at the same time as rotenone leads to significant cell survival in general, and particular of the ENS neurons. Our initial hypothesis that VEGF also has a neuroprotective effect on ENS neurons is thus clearly supported.

## 3. Discussion

We investigate for the first time in enteric neurons the expression of different VEGF receptors, as well as the neuroprotective effects of VEGF on rotenone-treated enteric neurons, which have already been described in CNS neurons.

VEGF is best known for its key role in vasculogenesis and angiogenesis, although neuroprotective, neuroregenerative and neuroplastic effects have also been described in both the CNS and PNS [17,19,33,34]. To date, however, there are no data on the expression of VEGF receptors and their functions in the ENS. The ENS in particular is currently being discussed in many studies as the origin of neurodegenerative diseases, which spread from there via the vagus nerve into the brain. Therefore, the current work provides interesting and important data on VEGF-mediate neuroprotection in the ENS.

### 3.1. VEGF-Receptors in the ENS

To study the effects of VEGF on enteric neurons, precise knowledge of the expression of the corresponding receptors at different age stages is first required. Our study shows an almost constant, age-independent expression of VEGFR-2 and its two co-receptors NRP1/NRP2 both at the mRNA level and, for the particularly important and most strongly expressed receptor VEGFR-2, also at the protein level. For possible therapeutic approaches via VEGF-mediated signaling pathways, the expression of the receptors in old age is crucial, since neurodegenerative diseases occur more frequently with increasing age. Studies in the CNS have shown a downregulation of VEGFR-2 expression in mature neurons, whereas VEGFR-1 was already less strongly expressed in young age stages [38]. Correlatively, application of VEGF promotes somato- and dendritogenesis in neonatal and juvenile, but not in mature CNS neurons [38]. Since neuroprotective effects of VEGF are primarily mediated by VEGFR-2, this could at least limit the therapeutic effect of VEGF administration in the CNS in the elderly [33,34].

In contrast, the age-independent expression of the various VEGF receptors in the ENS is a very good prerequisite for VEGF-mediated therapeutic neuroprotection. Here, for example, the well expressed NRP1 could further increase the potency of VEGFR-2 by enhancing the binding of VEGF-A-165 [47].

We found different temporal patterns for VEGFR-1 and VEGFR-3, although these could only be detected in mature ENS neurons by qRT-PCR. Similar results for neurons in the CNS were obtained for VEGFR-3, with its expression increasing steadily from the beginning of neuronal differentiation to the mature stage in many neurons [36]. In addition to its established role in early nervous system development, VEGFR-3 is likely to be important for the function of adult CNS neurons [36].

Further studies need to follow to accurately determine the expression of VEGFR-1 and VEGFR-3 throughout the gut at different ages. It is possible that the expression at young age stages is so low that the amount of cDNA we used is not sufficient for detection. Thus far, it is also unclear whether the receptor distribution varies in different intestinal segments and different parts of the ENS. This knowledge could also be interesting for the study of intestinal development, as VEGFR-1 alters the functionality of VEGFR-2 as its decoy receptor [19,20]. Patients with neurodegenerative diseases, for example, might show a different receptor expression pattern in enteric neurons than control patients. Furthermore, there is evidence that the existing receptors are limited in their size of action. Amyloid-β-peptides (Aβ), which are typical of Alzheimer’s disease, have anti-angiogenic effects by binding to VEGFR-2 and preventing VEGF binding [28]. Therefore, it is interesting to investigate whether an increased supply of VEGF, for example, can attenuate the progression of the disease.

### 3.2. Therapeutic Impact of VEGF in the ENS

Due to the neuroprotective and neuroregenerative properties already known from the CNS, there are also studies on the therapeutic use of VEGF in connection with neurodegenerative diseases. However, similar data and studies on the use of VEGF in the ENS are still lacking.

In order to investigate the neuroprotective and neuroregenerative potential of VEGF in the enteric nervous system, models of the pathogenesis of neurodegenerative diseases are needed.

The problem is that for most neurodegenerative diseases, such as Parkinson’s disease, the key causative mechanisms are unfortunately not yet known. However, it is known that prolonged exposure to substances that disrupt the mitochondrial respiratory chain, for example by damaging mitochondrial complex I, probably play a role in the development of idiopathic Parkinson’s disease [40]. Pesticides are a possible group of substances that trigger this. Studies show that increased oxidative stress correlates with a negative influence of pesticides on the mitochondria, and that these factors are clearly more frequent in Parkinson’s patients [44,46].

In the present studies, we chose the established Parkinson’s model, in which neurons were treated in vitro for 24 h with the pesticide rotenone at a concentration of 800 nM [41]. Rotenone inhibits mitochondrial complex I and is used as a good model for studying the pathogenesis of Parkinson’s disease [26,39,40,41,42,43,44,45]. As in other studies, these conditions showed significant cell toxicity, but without destroying all neurons [41]. In the ENS neurons treated with rotenone and VEGF at the same time, significantly fewer dying cells were seen, demonstrating for the first time the neuroprotective potential of VEGF in the ENS. Further studies need to clarify whether VEGF can also have an effect on neuroregeneration in the ENS, i.e., whether administration of VEGF leads to neuroregeneration after existing damage caused by rotenone. Studies with a prolonged, chronic exposure to lower amounts of rotenone could be of interest, since it is known that this leads to alpha-synuclein-positive cyto-plasmatic inclusions as well as specific affection of complex I and nigrostriatal dopaminergic neurons [39,40]. Since there is growing evidence that pathological changes in the gut occur well before the first signs of neurodegeneration in the CNS and that pathologies probably extend into the brain via the gut-brain axis [9], findings on neuroprotection and neuroregeneration in the ENS are of particular relevance. Changes in the neurons of the intestine can be detected relatively easily by biopsies during endoscopic examination [13]. Therefore, it is now to be recorded whether toxins, which are assumed to promote neurodegenerative diseases, also influence VEGF receptor expression in the gut. Such studies are important in order to be able to assess the therapeutic potential of VEGF in the ENS in a next step.

It is also interesting that in the present experiments not only neurons survived due to VEGF application, but VEGF also had a protective effect on the other cells. However, our experiments cannot clarify whether this is related to the high density of neurons in the cell culture system or whether in vivo studies with a meshwork of different cells in the intestinal tissue would come to the same conclusion. Whether VEGF, in addition to its neuroprotective effect on ENS neurons, also has an influence on the pathogenesis of Parkinson’s disease or other neurodegenerative diseases in general can also only be speculated on the basis of the available data and should be the subject of future studies. Another interesting target for further analysis would be the neurotoxin 6-hydroxydopamine, which plays a role in the development of Parkinson’s disease. In the CNS, its expression is suppressed by the administration of VEGF [27,48]. Whether this also applies to the ENS remains to be seen.

It should also be noted that VEGF administration is also likely to promote side effects such as an increase in vasculogenesis and angiogenesis [15]. In Alzheimer’s disease, this pro-angiogenic effect could be used positively in the CNS [28]. Vascular dementia due to lacunar infarcts associated with systemic small vessel disease could also benefit from increased VEGF [30]. However, vasculogenesis is also crucial for tumor growth and other diseases [15]. In colon cancer, increased VEGF expression correlates with tumor size, infiltration and metastasis rate [16], and in the pathophysiology of colitis and inflammatory bowel disease, VEGF also plays an important role. Here, the bacterium *Clostridium difficile* increases VEGF production in the colon to increase vascular permeability [49]. On the other hand, decreased VEGF expression leads to local ischemia of the intestinal wall and bacterial infestation, including subsequent intestinal wall necrosis [50]. One way to get around these problems could be the use of VEGF-A-165b or VEGF-B. Studies have already shown that VEGF-A-165b has neuroprotective and neurotrophic effects in the CNS and PNS, but no effects on angiogenesis, while VEGF-B has already been shown to have neuroprotective effects in a Parkinson’s disease model [24,25,26]. If VEGF is to be administered therapeutically in a neuroprotective and possibly also a neuroregenerative manner, undesirable side effects must first be recorded, investigated and then also taken into account in subsequent therapy planning.

## 4. Materials and Methods

### 4.1. Animals and Preparation of the Rat Small Intestine (p9, p15, p30)

All procedures were conducted under established standards of the German federal state of North Rhine Westphalia, in accordance with the European Communities Council Directive 2010/63/EU on the protection of animals used for scientific purposes.

The small intestine of Wistar rats of both sexes at the age of postnatal day (p) 9, 15 and 30 was dissected as previously described [49]. Animals were decapitated, the jejunum and ileum were removed without the attached mesentery, cut into approximately 15 cm long pieces and cleaned with cold RNase-free phosphate buffered saline (PBS, No. 18912-014, Gibco). Then, the full length of every piece was opened at the base of the mesentery and cut into smaller pieces of 2–3 cm using small scissor. With the serosa pointing downwards the pieces were placed on sterilized, aluminium foil wrapped Microscope slides (AAAA000001##12E, Thermo Scientific, Waltham, MA, USA) and snap frozen in −50 °C isopentane using the LIENS chamber [49]. The microscope slides with attached frozen tissue were stored in a 50 mL tube (No. 62.547.254, Sarstedt, Newton, NC, USA) at –80 °C for laser microdissection or at –20 °C for immunohistochemistry.

### 4.2. Cyosections of Rat Myenteric Plexus

Cryosections of the myenteric plexus were obtained using the flat frozen small intestine as described before [51]. For RNase-free conditions, removable parts of the cryostat (CryoStar NX50, Thermo Scientific) were cleaned with NaOH-EDTA (0.1 M NaOH, 1 mM EDTA). Cryosectioning was performed at a chamber temperature of −20 °C and a stage temperature of −18 °C to −16 °C. In the chamber, the frozen pieces of intestine were stabilized on the microscope slide with tissue freezing medium (No. 14020108926, Leica Biosystems, Wetzlar, Germany). After 20–30 min acclimation time, a small piece was attached to the object holder of the cryostat with the serosa now facing up. In this way, the intestine was cut layer by layer until the myenteric plexus could be seen between the circular muscle layer and the longitudinal muscle layer as already described before [51,52]. Then, 10 μm cryosections for immunohistochemistry were mounted on Superfrost-Plus Adhesion Slides (J1800AMNZ, Thermo Scientific) and stored at 4 °C until further use. For laser microdissection, cryosections were mounted onto RNase-free polyethylene naphthalate (PEN) membrane slides (1.4 mm) made for LMD (No. 11505151, Leica Microsystems, Wetzlar, Germany). If the cryosections were not lasered on the same day, they were frozen at −80 °C.

### 4.3. mRNA Expression Level Analysis

#### 4.3.1. Cresyl Violet Staining

Slices were stained under RNase-free conditions immediately before use. Based on the RNA handling protocol from Zeiss they were first fixed with 70% ethanol for 2 min. This was followed by application of fresh, filtered 0.5% cresyl violet solution (cresyl violet acetate, No. 7651.1, Carl Roth, Karlsruhe, Germany) in 50% ethanol for 20 s [53]. The excess paint was poured off on a cloth. The whole membrane slides were repeatedly dipped in 50% and 70% ethanol. Finally, the slides were dehydrated in 100% ethanol for 30 s and air-dried at room temperature.

#### 4.3.2. Laser Microdissecion

Laser microdissection was performed as described previously [51]. Leica LMD software (LMD650, Leica Application Suite (LAS), Leica Microsystems, Wetzlar, Germany) was used to mark the myenteric ganglia. Areas between 1000 μm^2^ and 20,000 μm^2^ were isolated using a Leica LMD6500 (No. 11505151, Leica Microsystems) and following system settings: power 34; aperture 17; speed 33; specimen balance 18; ×20 magnification (Figure 1). Larger pieces tend to get caught on the underside of the slide, smaller ones are inefficient to collect. As soon as 1 mm^2^ of myenteric ganglia was collected, 20 μL of RNA lysis buffer (Monarch^®^ Total RNA Miniprep Kit, T2010S, New England BioLabs, Ipswich, MA, USA) was added. Samples were centrifuged for 5 min at 10,000 rpm and stored at −80 °C until further use.

#### 4.3.3. RNA-Isolation

Total RNA-Isolation from LMD samples was performed according to the manufacturer’s protocol for cells from the Monarch^®^ Total RNA Miniprep Kit (T2010S, New England BioLabs). Therefore, 7 mm^2^ of collected myenteric plexus samples were thawed on ice, pooled in a 1.5 mL microtube and filled up to a total of 300 μL volume of lysis buffer for resuspension. Samples were transferred to the gDNA removal column and centrifuged. After 300 μL ethanol (≥95%) was added to the flow, the mixture was placed onto the RNA purification column. The flow-through was discarded. On-column DNase I treatment was done followed by three washing steps with 500 μL RNA priming buffer and centrifugation. On an RNase-free microfuge tube, 50 μL nuclease-free water was added to the column matrix and centrifuged again. The isolated RNA (~900 ng/μL) was used immediately or alternatively stored at −80 °C.

#### 4.3.4. cDNA-Synthesis 

We performed cDNA synthesis using GoScript Reverse Transcription Mix, Oligo(dT) (Thermo Fisher Scientific, Waltham, MA, USA). (A2790, Promega, Madison, WI, USA) following the manufacturer’s recommendations. Samples consisting of 4 μL nuclease-free water; 4 μL GoScript Reaction Buffer, Oligo(dT); 2 μL GoScript Enzyme Mix and 10 μL undiluted total RNA were incubated in 0.2 mL microtubes for 5 min at 25 °C, 60 min at 42 °C, 15 min at 70 °C and hold at 4 °C. If the samples were not immediately used for RT-qPCR, they were stored at −20 °C.

#### 4.3.5. qRT-PCR

With an amount/concentration of 400 ng/μL cDNA we performed qRT-PCR with GoTaq^®^ qPCR Master Mix (A6001, Promega) according to manufacturer’s instructions.

Expression levels for the genes of interest and for housekeeping gene GAPDH were measured in triplicates in three independent PCR runs using the CFX96 Real Time PCR Detection System (BioRad, Hercules, CA, USA)**.** The following primers were used: FLT1 (codes for VEGFR-1): (5′-GTG AAG AGT GGG TCG TCA TTC-3′, 3′-CTA TGG TTT CCT GCA CCT GTT-5′, Microsynth, Göttingen, Germany); KDR (codes for VEGFR-2): (5′-TCC CAG AGT GGT TGG AAA TG-3′, 3′-ACT GAC AGA GGC GAT GAA TG-5′, Microsynth); FLT4 (codes for VEGFR-3): (5′-CTG GAC ACC CTG TAA GAC ATT T-3′, 3′-AGT GGT CAC CTC CTT CCA-5′, Microsynth); NRP1: (5′-GCA TCC TGG GAA ACT GGA ATA-3′, 3′-GCT GTA ATC TGG GAG TCT GAA A-5′, Microsynth); NRP2: (5′-GGC TTC TCA GCA CGT TAC TAT T-3′, 3′-TGA GGC ACT GAT CTG TTC ATT AG-5′, Microsynth) and the housekeeping gene *GAPDH*: (5′-ACT CCC ATT CTT CCA CCT TTG- 3′, 3′-CCC TGT TGC TGT AGC CAT ATT-5′, Microsynth). Quantitative RT-PCRs were performed on a CFX Connect Real Time PCR Detection System (Bio-Rad, Hercules, CA, USA). Microsoft Excel (Microsoft Corporation, Redmond, WA, USA) and GraphPad Prism Version 7.0a (GraphPad, San Diego, CA, USA) were used for statistical analysis. The 2^−ΔΔct^ method was applied, using the geometric mean and normalized to the housekeeping gene GAPDH, then the results were plotted logarithmically to base 10.

### 4.4. Immunohistochemistry on Cryosections

For immunohistochemical staining, the cryosections were first bordered with Pap Pen (MKP-2, Kisker, Steinfurt, Germany), fixed with paraformaldehyde (PFA) in PBS for 15 min and rinsed three times with PBS for 5 min. The slices were permeabilized using 1% Triton-X-100 (T8532, Sigma-Aldrich, St. Louis, MO, USA) in PBS for 15 min. Following three washing steps with PBS for 2 min, goat serum (G9023, Sigma-Aldrich; 1:50 in PBS) was added for 30 min to block non-specific binding sites. After a short 2 min washing step with PBS, the neuron-specific primary antibody PGP 9.5 (pan-neuronal marker, chicken, PA1-10011, Thermo Fisher; 1:200 in 1:50 goat serum in PBS) or beta-III-tubulin (TUJ-1, mouse, MAB1195, RD-Systems, Bio-Techne, Minneapolis, MN, USA; 1:500 in PBS) (supplementary figure) was applied overnight at 4 °C. The next day slices were rinsed with PBS three times for 2 min and twice for 10 min. Then, secondary antibodies, either anti-chicken IgY (goat, A11041, Invitrogen, Waltham, MA, USA; 1:400 in PBS) when using PGP 9.5 as primary antibody or anti-mouse IgG (goat, T5393, Sigma-Aldrich; 1:750 in PBS) for beta-III-tubulin were applied on the slices for 2 h at room temperature. Again slices were rinsed with PBS three times for 2 min and two times for 10 min before slices were incubated with primary antibodies against VEGFR-2 (rabbit, ab5473, Abcam, Cambridge, UK; 1:500 in PBS) at 4 °C overnight. Before and after application of secondary anti-rabbit IgG antibodies (donkey, A-21206, Invitrogen; 1:750 in PBS) for 2 h at room temperature slices were rinsed with PBS three times for 2 min and two times for 10 min. Nuclear counterstaining was performed with DAPI (D9542, Sigma-Aldrich; 1:5000 in PBS) for 30 min. After washing three times for 2 min and two times for 10 min with PBS slices were embedded in fluorescence mounting medium (S3023, Dako, Glostrup, Denmark) and stored at 4 °C until use.

### 4.5. Cellculture

Myentericus plexus cell cultures from p15 Wistar rats were prepared as described before [51,54]. Succinctly, the small intestine from three rats was removed as explained in 4.1, stored in cooled MEM-P/S (MEM-Hepes (M7278, Sigma-Aldrich) containing 1% penicillin-streptomycin (p4333, Sigma-Aldrich) and cut into 3 cm long pieces. To separate the transparent tunica muscularis from the rest of the intestine, one piece at a time was stretched on a thin rod (e.g., Combitip Plus tip, p1877, Eppendorf, Hamburg, Germany). Using the curved edge of a forceps the entire outer layer of the intestine was carefully loosened and pulled at once.

In 1.5 mL tubes each filled with 1.3 mL enzymatic digestion mix (HBSS (H9269, Sigma-Aldrich) containing 1% penicillin-streptomycin (p4333, Sigma-Aldrich); 0.75 mg/mL Liberase (No. 5401135001, Sigma-Aldrich); 20 mg/mL Deoxyribonuclease I (No. 002139, CellSystems, Troisdorf, Germany)), very small pieces of the tunica muscularis were enzymatically digested for 4 h at 37 °C in the Rollodrum (New Brunswick Scientific Co., Inc., Enfield, CT, USA). The tubes were manually shaken once every hour and at the end of the incubation period. After centrifugation at 775 rpm for 5 min, the supernatant was discarded. The fluid from two tubes containing the myenteric neurons was collected in 35 mm Petri dish (No. 83.3900, Sarstedt, Newton, NC, USA). To remove residues of the enzymatic digestion mix, 2 mL of MEM-P/S were added and the supernatant was removed again after 1 min. This process was repeated once more before 400 μL of proliferation medium (50 mL proliferation medium contains: 47.875 mL Neurobasal™-A Medium (No. 10888022, Thermo Fisher); 0.125 mL Glutamine (G7513, Sigma-Aldrich); 0.5 mL fetal bovine serum (F7524, Sigma-Aldrich); 0.5 mL Penicillin-Streptomycin (P4333, Sigma-Aldrich); 1 μL FGF-2 (SRP4039-50UG, Sigma-Aldrich) ≙ 20 ng/mL; 0.5 μL EGF (SRP3238-100UG, Sigma-Aldrich) ≙ 10 ng/mL; 1 mL B-27™ Supplement (50×), minus vitamin A (No. 12587010, Thermo Fisher)) was added. The content of three 35 mm Petri dishes was split into 24 wells, each containing 500 μL proliferation medium. After three days of cultivation at 37 °C and 5% CO_2_, the medium was exchanged.

**Figure 4:**
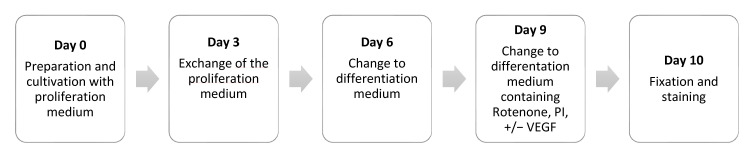
Timeline for the cell culture of myenteric plexus neurons.

On day 6, the proliferation medium was replaced by 150 μL differentiation medium. (50 mL differentiation medium contains: 47.375 mL Neurobasal™-A Medium (No. 10888022, Thermo Fisher); 0.125 mL Glutamine (G7513, Sigma-Aldrich); 0.5 mL fetal bovine serum (F7524, Sigma-Aldrich); 0.5 mL Penicillin-Streptomycin (P4333, Sigma-Aldrich); 1 mL Neuromix 2a plus retinoic acid (No. 17504044, Thermo Fisher); 0.5 mL Mitosis-inhibitor (1 mM 5-Fluoro-2′Deoxyuridin (21555, Serva Electrophoresis GmbH, Heidelberg, Germany); 1 mM Cytosin-βD-Arabinofuranosid (C6645, Sigma-Aldrich); 1 mM Uridine (u3003, Sigma-Aldrich)) and the cells from two wells were transferred onto a coverslip coated with Poly-D-Lysine (P7280, Sigma-Aldrich) in a 12-well plate. Another 200 μL of differentiation medium were filled into each well before further incubation at 37 °C and 5% CO_2_ for three days. Figure 4 provides an overview of the chronological sequence of the cell culture.

### 4.6. In Vitro Treatment with Rotenone +/− VEGF

After three days of differentiation, the medium was exchanged with differentiation medium containing 800 nM Rotenone (No. 45656, Sigma-Aldrich). The 0.1 mM stock solution of rotenone was stored in the freezer for a maximum of one month. To investigate the effects of VEGF on the neurons, a concentration of 0.1 μg/mL VEGF (V4512, Sigma-Aldrich) was used in half of the wells. This concentration has already been used for in vitro studies on CNS cells [38]. From now on every step was performed with as little light as possible. Each well was additionally filled with 0.5 μL propidium iodide (PI; 5 μg/mL; P4170, Sigma-Aldrich) to mark dead cells. The cells were then incubated for 24 h.

### 4.7. Immunohistochemical Analysis of ENS Cultures

Following incubation cells on glass coverslips were fixed and stained almost exactly as described above (4.4). However, unlike for the cryosections, a concentration of 0.3% Triton-X-100 was used for these cells. Furthermore, work must be carried out without significant exposure to light. Neurons were labelled with beta-III-tubulin (TUJ-1, MAB1195, RD-Systems; 1:500 in PBS) and its secondary anti-mouse antibody (goat, A11001, Molecular Probes, Eugene, OR, USA; 1:1000 in PBS) before counterstaining with DAPI was performed. The total cells (nuclei stained with DAPI), the beta-III-tubulin positive neurons (green) and the proportion of dead cells marked with propidium iodide (red) were counted with the aid of confocal laser scanning microscopy (CLSM, LSM 800, Carl Zeiss, Jena, Germany).

To ensure blinded conditions, each slide was labeled with a cover slip by a person with a combination of two letters and two numbers. The test group (−/+ VEGF) and the exact test data of the cover slips were recorded together with this random name in an Excel document. This document was not accessible during the analysis of the photos. A different person took photos at the LSM, counted the cell groups and collected the results in another Excel file. Only after all cover slips had been photographed and analyzed all files were combined. Following categories were formed for the control and for the VEGF-treated cultures: all cells, all neurons, dead cells, and dead neurons. For both conditions (−/+ VEGF), 24 coverslips each were evaluated from 3 independent cell cultures.

## Data Availability

Not applicable.

## Supplementary Materials

The following supporting information can be downloaded at: https://www.mdpi.com/article/10.3390/ijms23126756/s1.
