# Supplementary material for: Neuroprotective Effects of VEGF in the Enteric Nervous System
Source: Int J Mol Sci. 2022 Jun 17;23(12):6756. doi: 10.3390/ijms23126756 (PMC9224388; doi:10.3390/ijms23126756)
Supplement: Supplementary file 1 [file ijms-23-06756-s001.zip › ijms-1753828-supplementary.pdf]

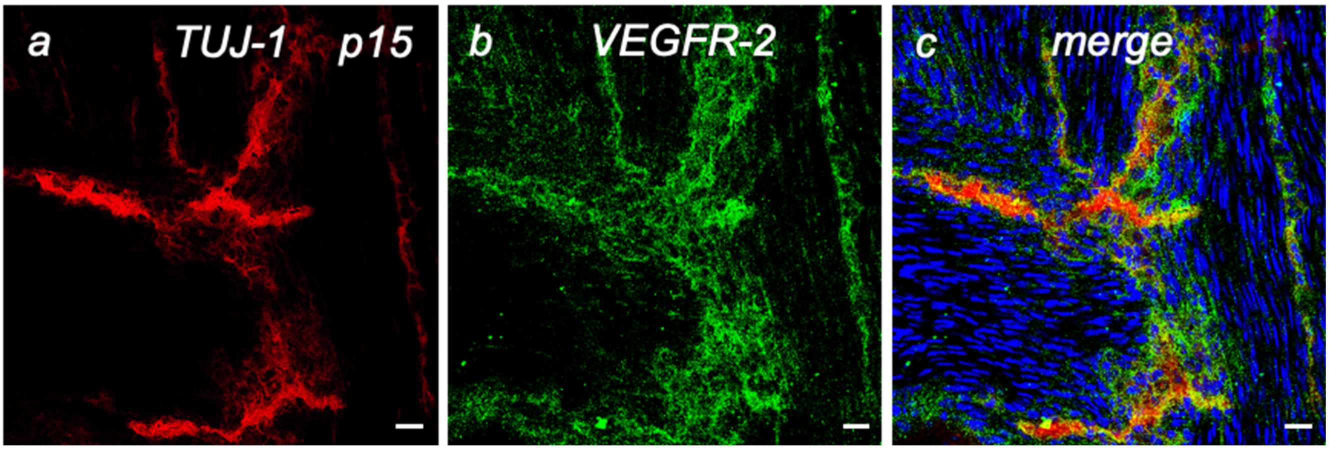

Figure S1. (**a**–**c**) Exemplary immunostaining of TUJ-1-positive neurons at p15, VEGFR-2 (green) and cell nuclei (blue) also show the colocalization of VEGFR-2 and myenteric neurons. The same exposure settings were used for all exposures. Scale bars: 20 μm.
